# F219. NOVEL OBJECTIVE ASSESSMENT OF ACTIVITY ENGAGEMENT IN SCHIZOPHRENIA USING WIRELESS MOTION CAPTURE

**DOI:** 10.1093/schbul/sby017.750

**Published:** 2018-04-01

**Authors:** Ishraq Siddiqui, Gary Remington, Gagan Fervaha, Paul Fletcher, Aristotle Voineskos, Sarah Saperia, Konstantine Zakzanis, George Foussias

**Affiliations:** 1 Centre for Addiction and Mental Health; 2 University of Toronto Scarborough

## Abstract

**Background:**

Amotivation and reduced engagement in goal-directed activities are prominent features of schizophrenia. Previous investigations of patients’ engagement in activities have largely relied on accounts of daily living activities rather than objective task-based measures. The current study used wireless motion capture in an open-field setting to evaluate activity preference when individuals are provided an explicit choice between an active engagement option versus a passive engagement option.

**Methods:**

Twenty stable adult outpatients with schizophrenia and twenty matched healthy controls completed the Activity Preference Task, in which participants play a physical motion-based video game (active engagement) or watch a film (passive engagement) for fifteen minutes. No incentive was associated with either activity, and participants could engage in either activity at any time. Duration of engagement on the active option and number of switches between activity options were computed as the primary task outcome measures using objective motion data. Participants’ behaviour during active engagement was further quantified by computation of physical intensity (average hand speed) and persistence (tendency for sustained continuous engagement). Clinical assessments of positive and negative symptoms, apathy, cognition, depression, medication side-effects, motor ability, and community functioning were also administered.

**Results:**

Schizophrenia participants’ duration, intensity, and persistence of active engagement were correlated with apathy (|ρ|=0.72–0.79, p<0.01) and community functioning (ρ=0.50–0.67, p<0.05). Although no significant group differences were detected in the individual comparisons of task measures, exploratory cluster analysis based on the two primary task measures identified three clusters of individuals with distinct profiles of engagement intensity (F(2,36)=9.141, p<0.001) and persistence (F(2,36)=13.954, p<0.001), and clinical apathy (F(2,37)=4.183, p=0.023). Further, there were significant diagnostic group by cluster assignment interaction effects for engagement intensity (F(2,33)=4.551, p=0.018) and apathy (F(2,34)=3.445, p=0.043) that highlighted substantial behavioural heterogeneity specific to schizophrenia; these interaction effects appeared to be driven primarily by a subgroup of patients who exhibited reduced engagement and increased apathy compared to individuals in other clusters as well as within-cluster healthy control counterparts.

**Discussion:**

The Activity Preference Task provides a means of quantifying activity engagement in schizophrenia, which may be particularly valuable given the lack of objective assessments that measure non-incentivized, intrinsically motivated behaviours. Our initial findings suggest that patients with schizophrenia as a group are equally inclined as healthy individuals towards actively engaging activities when presented an explicit choice, but provision of such opportunities may be insufficient for amotivated patients to initiate and maintain engagement in functional behaviours.

